# Glass ionomer ART sealant and fluoride-releasing resin sealant in fissure caries prevention – results from a randomized clinical trial

**DOI:** 10.1186/1472-6831-14-54

**Published:** 2014-05-19

**Authors:** Bao Ying Liu, Yue Xiao, Chun Hung Chu, Edward Chin Man Lo

**Affiliations:** 1 Faculty of Dentistry, The University of Hong Kong, 34 Hospital Road, Hong Kong, SAR, China

**Keywords:** Fissure sealants, Glass-ionomer cement, Child, Dental caries, Dental fissures, Preventive dentistry, Atraumatic restorative treatment

## Abstract

**Background:**

The relative performance of ART sealant and fluoride-releasing resin sealant in preventing fissure caries in permanent molars was compared in a randomized clinical trial conducted in southern China (ClinicalTrials.gov NCT01829334).

**Methods:**

After obtaining ethical approval, healthy schoolchildren who had permanent first molars with occlusal fissures which were sound but deep or presented with only incipient caries were recruited for the study. Included molars were randomly allocated into one of four parallel study groups in units of left/right teeth per mouth. Two of the four groups adopted the methods of ART or fluoride-releasing resin sealant placement while the other two groups adopted the topical fluoride application methods. Fissure status of the molars in each group was evaluated every 6 months. Development of dentine caries and sealant retention over 24 months in the molars in the two sealant-using groups was compared in this report. Outcome on cost-effectiveness of all four groups over 36 months will be reported elsewhere.

**Results:**

At baseline, a total of 280 children (383 molars) with mean age 7.8 years were involved for the two sealant groups. After 24 months, 261 children (357 molars) were followed. Proportions of molars with dentine caries were 7.3% and 3.9% in the ART sealant and fluoride-releasing resin sealant groups, respectively (chi-square test, p = 0.171). Life-table survival analysis showed that sealant retention (full and partial) rate over 24 months for the resin sealant (73%) was significantly higher than that (50%) for the ART sealant (p < 0.001). Molar survival (no development of dentine caries) rates in the ART sealant (93%) and fluoride-releasing resin sealant (96%) groups were not significantly different (p = 0.169). Multilevel logistic regression (GEE modeling) accounting for the effects of data clustering and confounding factors confirmed this finding.

**Conclusions:**

Though the retention of fluoride-releasing resin sealant was better than that of the ART sealant, their effectiveness in preventing fissure caries in permanent molars did not differ significantly over 24 months. ART sealants could be a good alternative when and where resources for resin sealant placement are not readily available.

## Background

Pits and fissures in the first molars are the most susceptible sites for dental caries in the permanent dentition [1,2] and contemporary studies show specifically that 85% or more of the caries is nested in the above-mentioned sites [3,4]. Thus, prevention of caries in these tooth sites is of crucial importance in keeping a sound permanent dentition.

Sealing the pits and fissures of molars and premolars for prevention of dental caries was first introduced in the 1960s [5]. It is now accepted as a highly effective method in preventing dental caries [6]. The two predominant types of dental sealant nowadays are resin-based and glass ionomer cement (GIC) sealants. The effectiveness of resin sealant in preventing fissure caries depends primarily on its retention after placement [6,7]. For high-quality resin sealant placement, electrically powered dental equipment and good clinical conditions are required. However, this may be difficult to achieve in places where access to a modern dental clinic is limited. This problem may be overcome by using GIC sealants because they can be placed without the use of electrically powered dental equipment.

Although a number of different types of glass ionomers have been used previously as sealants, largely low-viscosity Type III sealants and ART sealants using high viscous type II restorative GIC, systematic reviews comparing GIC sealants with resin sealants have almost always, perhaps unfairly, pooled these different types of GIC sealants under a single category which makes true specific comparisons difficult. Consequently, inconsistent findings have been found regarding the comparative effectiveness of GIC to resin sealant in preventing fissure caries [6,8] and there is no clear evidence to support the superiority of either of the two types of sealants [9,10]. However, there is a wide range of GIC materials available in the market with different formulations, properties, and performance for use in dentistry.

Strengthened highly viscous (type II restorative) GIC has the property of rapid setting, considerably reduced moisture sensitivity in the early setting stage and low solubility in oral fluids [11] which make it an improved GIC material for the atraumatic restorative treatment (ART) technique [12]. The latest particle formula of such kind of GIC material improved its wettability leading to an easier and faster mixing for practical use. Placement of ART sealant uses such a kind of GIC material to seal pits and fissures with the aid of a similar “finger-press” technique as used in the ART restoration procedure. It was found that retention rate of ART sealants was higher than that of the earlier developed lower viscosity ones [13,14]. The success rate in preventing fissure caries over 6 years by using ART sealant can be as high as 85% [15]. A study found that ART sealant could outperform resin sealant in fissure caries prevention [16]. It seems promising for ART sealant to be used as an alternative to resin sealants. However, at least until the end of 2010, well conducted clinical/field trials demonstrating the relative effectiveness of ART sealant and that of resin sealant are still limited, yielding insufficient evidence to draw a conclusion on the comparison between the two [17]. More studies are therefore needed to document the possible differences between resin and ART sealants in their effectiveness in fissure caries prevention.

A randomized controlled trial aimed at comparing the effectiveness of four different methods in preventing pit and fissure caries in permanent molars was conducted in southern China. The aim of this article was to report on the 24-month results of this trial, targeting specifically on the relative effectiveness of a self-cured ART sealant and that of a light-cured fluoride-releasing resin sealant in fissure caries prevention in permanent first molars of schoolchildren. Results on long-term cost-effectiveness of all four groups over a 36-month period will be reported elsewhere. The null hypothesis to test in this report was that there was no difference between the effectiveness of the two types of fissure sealants.

## Methods

This study was a randomized clinical trial with four parallel groups (ClinicalTrials.gov NCT01829334). Besides the two sealant-adopting groups compared here, the other two groups involved fluoride application. Ethical approval of the study was obtained from the Institutional Review Board of the University of Hong Kong. It was carried out in Shenzhen, China where the water supply was not fluoridated but fluoride toothpaste was common in the market. The concurrent dental caries prevalence of the 12-year-old children in Shenzhen when this study was started was 29.8% (mean DMFT score: 0.54) and 66.7% of the dental caries was nested in the permanent first molar [18].

Children aged 7 to 9 years attending two of the largest primary schools in Shenzhen were invited to participate in the study. Children with written parental consent were clinically examined by dentists in their school. Children who did not have any major general health problems and had permanent first molars with occlusal fissures which were deep (base of fissure cannot be seen) or presented with signs of incipient caries (opacity and discoloration seen when viewed wet), similar to ICDAS code 2 [19] were included. Children who were uncooperative or refused dental treatment were excluded. Molars fulfilling the above requirements in the recruited children were visually assessed by using an intra-oral LED light and disposable mouth mirrors to record their baseline status (1-no caries, deep fissures; 2-fissures with signs of incipient caries). CPI probes were used to remove plaque obscuring visual assessment when necessary. The molars were also assessed by DIAGNOdent 2095 (KaVo Dental, Biberach, Germany), a laser-induced fluorescence based caries detection device [20]. DIAGNOdent measurement was repeated three times for each molar and the highest reading was recorded. In this study, DIAGNOdent readings ≥40 were taken to indicate that the screened molars potentially had dentine caries [21]. Molars presented with DIAGNOdent reading ≥40, carious cavities, dental sealant, fillings, and/or hypoplasia were excluded. The chief examiner (YX) of this study and another dentist from a local public hospital were involved in the baseline examination. They received training on the use of the DIAGNOdent device and the diagnosis criteria (on dentine caries, deep occlusal fissure, incipient caries, as well as sealant retention evaluation) used in this study. They were calibrated before the start of this study with an experienced epidemiologist on a group of selected child patients in the dental clinic of the local hospital.

Included molars were randomly allocated into one of four parallel study groups (resin sealant, ART sealant, and two topical fluorides) in units of left/ right teeth per subject. If molars of both sides of one mouth were included, the molars on one side of the mouth were assigned to one group and those on the other side were assigned to another group. If only molars on the same side of one mouth were included, only one group was assigned to the mouth. At least one and at most two groups would be assigned in the same mouth. Two groups using topical fluorides would not be assigned in the same mouth. There were five possible combinations of two out of the four study groups (resin sealant/ART sealant, resin sealant/SDF - silver diammine fluoride solution, resin sealant/NaF - sodium fluoride varnish, ART sealant/SDF, ART sealant/NaF) and these were assigned with numbers 1 to 5. Papers with the numbers written on were put into an envelope to be drawn by an assistant to decide the group combination of the included molars of a subject. A coin was then thrown to decide which side of the molars would be assigned the group with a smaller group number in the combination. If only molars of one side were included, the other group in the selected combination would be discarded.

Molars allocated in the two sealant groups received single placement of the fluoride-releasing resin sealant (Clinpro, 3 M ESPE, Seefield/Oberbay, Germany) or ART sealant (Ketac-Molar Easymix, 3 M ESPE, Seefield, Germany). Oral hygiene instruction was provided to all children in the study at baseline. The oral health related behaviors of the children including the toothbrushing habits and the frequency of taking sweet snacks and drinks were asked and recorded by the examiners/assistants.

Sealants were provided in the schools by four operators with help from chair-side assistants. The operators were two independent dentists from a local public hospital who carried out most of the sealant placement and the two aforementioned examiners who provided a small proportion of the treatments when the two independent dentists were not available. The chief examiner (YX) had good previous experience in providing the treatment while the other three dentists had little experience. Hands-on training on providing the two types of sealant was provided to the less experienced operators by YX in a compromised clinical environment before the start of this study. In sealant placement, the molar was isolated with cotton rolls. Occlusal surfaces of molars in resin sealant group were etched with 37% phosphoric acid for 15–20 seconds, washed with water which was removed by suction connected to a portable dental unit, and then dried with air blow from a 3-in-1 syringe attached to the dental unit. Resin sealant was then applied and light-cured for 20 seconds using a LED curing-light. Occlusal surfaces of molars in ART sealant group were conditioned with the liquid component of the glass ionomer for 10–15 seconds, cleaned by cotton pellets soaked with water, and then dried with cotton pellets. GIC material was hand mixed according to the manufacture’s instruction and placed using the finger-press technique [22]. Complete setting and retention of sealants and occlusion were checked before the children left.

Development of dentine caries (ICDAS Code 4–6) and sealant retention (completely retained, partially retained, and no sealant) in the molars was assessed blindly every 6 months by the same two calibrated dentists involved in the baseline examination. Intra-oral LED light, disposable plane front-surface mouth mirrors were used in the examinations. CPI probes to aid the diagnosis of dental caries and sharp sickle-shaped dental explorers to aid the assessment of sealant retention were used when necessary. Fissures with fully retained sealants were regarded as sound. The primary outcome was development of dentine caries in the study molars.

A 10% random sample, selected one out of every 10 subjects using systematic random sampling method by the dental assistant, was re-examined during every examination (baseline and follow-up). Inter-examiner reproducibility between the two examiners in this study during baseline examination and follow-up evaluation were monitored.

In sample size calculation, it was anticipated that around 90% of molars receiving resin sealant would not have dentin caries after 24 months [23]. To detect a clinically significant 10% absolute difference between two groups by using chi-square test based on α = 0.05 and 80% power, a total of 288 molars for two groups was required. With estimation of intraclass correlation (ICC) among the molars to be 0.1 and on average two molars were expected to be included in each child (i.e., m = 2), the calculated number was raised to 318 [24]. To allow for an overall 15% drop-out rate, a sample size of 376 molars in total for two groups was required at baseline.

### Data analysis

Data was input into computer and analysis was performed using SPSS 20.0 (SPSS Inc., Chicago, IL, USA). Chi-square test was used to compare the caries incidence rates. Life-table survival analysis was used to compare the cumulative molar survival (no dentine caries developed) rates and sealant retention (full and partial) rates over 24 months of the two groups. Considering the relatively short observation period and the low caries progression rate, a multi-level logistic regression analysis using generalized estimating equation (GEE) modeling which used single-time-point outcome data was also performed. Outcome was reported at the tooth level, and a two-level structure (level 1 - tooth; level 2 - child) was adopted. The dependent variable was presence of dentine caries at the 24-month examination. Independent variables included those at the child level: gender (boy/girl), age, snacking (≥2 times daily or not), toothbrushing (≥2 times daily or not); as well as variables at the tooth level: treatment (resin sealant/ART sealant), status at baseline (sound or present with signs of incipient caries), molar location (upper/lower), baseline DIAGNOdent reading (≤15/16-39) of the molar, group combination during random allocation (sealant/sealant, sealant/SDF, sealant/NaF) and period of sealant retention (0–4 times 6 months). Interaction effects between and among the independent variables were considered. Exchangeable and independent correlation structure of the clustering of molars in each child was also assessed, and the model yielding the lowest adjusted quasi-likelihood under the independence model criterion (QIC) value was selected as the final model.

## Results

**Figure 1:**
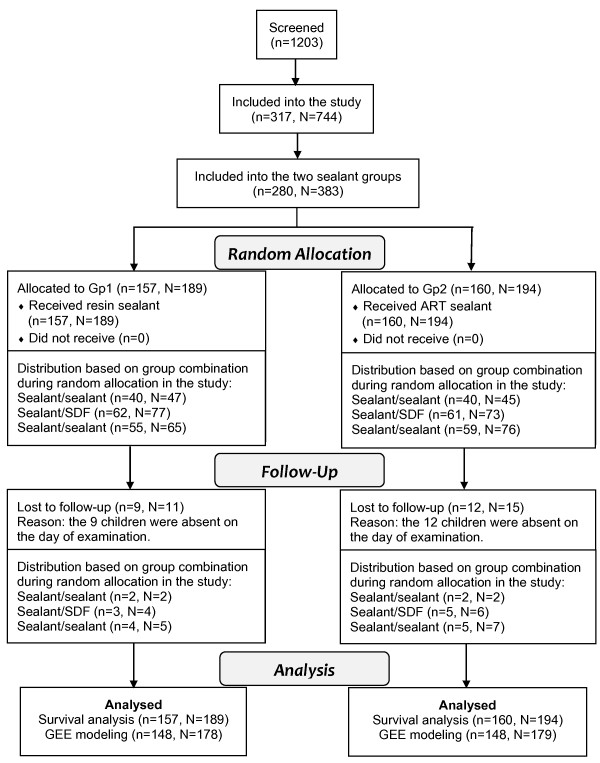
Subjects flow until 24 months in the two sealant groups in the study (n- number of subject, N-number of molar).

Among the 1203 children screened, a total of 317 children (45.7% boys) with 744 molars were recruited for this study. At baseline, a total number of 280 children (44% boys) with 383 permanent first molars were included into the two sealant groups in the study (Figure 1). Mean age of these children was 7.8 years. No statistically significant differences were found between the involved children for the two groups regarding their background and oral health behaviors as well as the distribution of included molars, except that a higher proportion of molars in the ART sealant group than in the resin sealant group had signs of incipient caries (19.6% vs. 12.2%, p = 0.047) (Table 1). The Kappa values of inter-examiner agreement in the baseline dental examinations and follow-up evaluations, including the development of dentine caries, were all greater than 0.88.

**Table 1:** Comparison of the baseline factors between the two groups

**Factors**	**Group**		p-value*
	**Resin sealant**	**ART sealant**	
**Participant**			
^#^Age (SD)	7.8 (0.66)	7.8 (0.66)	0.695
Gender			0.881
-Boy	70 (44.6%)	70 (43.8%)	
-Girl	87 (55.4%)	90 (56.2%)	
Snacking habit			0.152
-Once a day or less	116 (73.9%)	129 (80.6%)	
-Twice or more a day	41 (26.1%)	31 (19.4%)	
Tooth brushing habit			0.901
-Once a day or less	56 (35.7%)	56 (35.0%)	
-Twice or more a day	101 (64.3%)	104 (65.0%)	
Group allocation combination			0.942
-Sealant/sealant	40 (25.5%)	40 (25.0%)	
-Sealant/SDF^∆^	62 (39.5%)	61 (38.1%)	
-Sealant/NaF^Φ^	55 (35.0%)	59 (36.9%)	
**Molar**			
Group allocation combination			0.624
-Sealant/sealant	47 (24.9%)	45 (23.2%)	
-Sealant/SDF	77 (40.7%)	73 (37.6%)	
-Sealant/NaF	65 (34.4%)	76 (39.2%)	
Baseline molar status			**0.047**
-Sound with deep fissure	166 (87.8%)	156 (80.4%)	
-Present with incipient caries	23 (12.2%)	38 (19.6%)	
Location			0.964
-Upper molar	53 (28.0%)	54 (27.8%)	
-Lower molar	136 (72.0%)	140 (72.2%)	
DIAGNOdent reading			0.543
-0 ~ 15	77 (40.7%)	85 (43.8%)	
-16 ~ 39	112 (59.3%)	109 (56.2%)	

Comparisons were performed by using Chi-square test otherwise specified while respective percentage was reported in the parenthesis. ^#^Independent samples t-test was performed for the comparison while standard deviation (SD) of the respective data was reported in the parenthesis. ^∆^Silver diammine fluoride solution. ^Φ^Sodium fluoride varnish. *Statistical significance level is set at 0.05 and value of statistical significance is emphasized in bold.

A total of 261 (93.2%) children with 357 (93.2%) molars were followed at 24 months for the two sealant groups. There was no statistically significant difference between the two groups in the distribution of children and molars lost to follow-up. No statistically significant differences were found between the children followed at 24 months and those lost to follow-up regarding their background and oral health behaviors as well as the distribution of the molars. No complaints from the children and no adverse effect of the treatments were found during this study.

**Figure 2:**
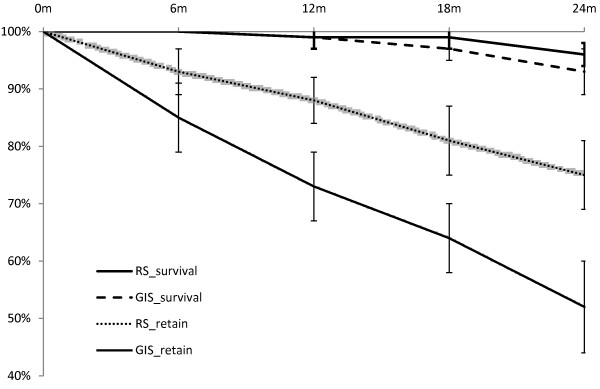
Cumulated proportions of molar survival and sealant retention over 24 months in the two sealant groups, resin sealant (RS) and ART sealant (GIS) (error bars show the 95% confidence intervals of the estimated mean).

The proportions of study molars with dentine caries at the 24-month examination in the resin and ART sealant groups were 3.9% (7 out of 178) and 7.3% (13 out of 179) respectively (p = 0.171). Results of the Life-table survival analysis showed that the cumulative molar survival (no dentine caries developed) rates over 24 months in the resin sealant and ART sealant groups were 96% and 93% respectively (p = 0.169). The proportions of sealants retained at the 24-month examination in the resin and ART sealant groups were 78.7% (140 out of 178) and 55.3% (99 out of 179) respectively (p < 0.001). The cumulative sealant retention (full and partial) rate over 24 months was 75% for the resin sealant and 52% for the ART sealant (p < 0.001). The mean number of survival periods (in units of 6 months) were 3.4 (SD = 1.2) and 2.7 (SD = 1.5) for the resin and ART sealants, respectively (p < 0.001). Molar survival (no dentine caries developed) and sealant retention in the two groups over the 24-month period of this study is shown in Figure 2. GEE modeling confirmed that the risk to develop fissure caries of molars receiving ART sealant did not significantly differ from that of molars receiving resin sealant in this study (Table 2). It was also found that the longer the retention time of the sealant on the molar, the lower the risk of developing dentine caries in the occlusal surfaces of the molar (OR = 0.453, p < 0.001), and that presence of incipient caries in the fissures before the placement of sealant would increase the risk of dentine caries development (OR = 4.662, p = 0.008).

**Table 2:** Full model of the 2-level GEE logistic regression (n_subject_ = 261, N_molar_ = 357)

**Factors**	**Estimation (S.E.)**	p-value*	**Odds ratio (OR**^**#**^**)**	**95% C.I. for OR**	
				**Lower**	**Upper**
Sealant (resin vs. ART sealant)	0.138 (0.613)	0.822	1.148	0.345	3.820
Age	−0.216 (0.508)	0.607	0.770	0.285	2.084
Gender (boy vs. girl)	−0.743 (0.678)	0.273	0.476	0.126	1.798
Candy snacking habit (<2 vs. ≥2daily)	−0.073 (0.596)	0.903	1.075	0.335	3.456
Tooth brushing habit (<2 vs. ≥2daily)	1.213 (0.771)	0.115	3.363	0.743	15.231
Incipient caries at baseline (yes vs. no)	1.432 (0.646)	**0.027**	4.187	1.180	14.854
Molar location (lower vs. upper)	0.232 (0.702)	0.742	1.261	0.318	4.922
DIAGNOdent reading (16–39 vs. ≤15)	1.930 (1.029)	0.061	6.890	0.917	51.778
Sealant retention (0–4 half-year periods)	−0.801 (0.188)	**<0.001**	0.449	0.310	0.648
Group allocation combination	-	0.544	-	-	-
-Sealant/SDF^∆^ vs. sealant/sealant	0.701 (0.853)	0.411	2.015	0.379	10.714
-Sealant/NaF^Φ^ vs. sealant/sealant	0.903 (0.818)	0.270	2.467	0.496	12.265
Intercept	−2.304 (4.350)	0.596	0.100	0.000	504.044

^#^Odds Ratio (OR) is the ratio of the odds to develop dentine caries under two compared conditions. ^∆^Silver diammine fluoride solution. ^Φ^Sodium fluoride varnish. *Statistical significance level is set at 0.05 and value of statistical significance is emphasized in bold.

## Discussion

Regarding the design of the study, cross-over effect of fluoride on the effectiveness of sealant placement in fissure caries prevention might exist, the amount of which cannot be estimated in this study. Despite this, similar proportions of molars in the two sealant groups were exposed to a topical fluoride application method in the same mouth. In addition, both types of sealant used in this study are fluoride-releasing materials. Thus, the above mentioned factor was considered to be balanced between the two sealant groups. Such factor was considered as a confounding factor and included in the logistic regression modeling during data analysis. The results show that it did not have a significant effect on the outcome of this study. Accumulation of the caries-preventive effect may be raised when combined application of different topical fluoride regimes are provided [25]. The fluoride released from the two sealants plus the parallel exposure to topical fluoride applied on the contra-lateral molars in the majority of the mouth may increase the caries-preventive effect of both the sealants. In this case, the difference in caries-preventive effect between the two sealants would become smaller and require a larger sample size to detect it. It should be noted that the small magnitude of difference, most likely less than 10%, may not be clinically significant.

In this study, attrition bias due to the subjects and molars lost to follow-up was considered not a major problem because the following two reasons. Firstly, no statistically significant differences were found between the children followed at 24 months and those lost to follow-up regarding their background and oral health behaviors as well as the distribution of the molars. This indicates that the subjects and the molars lost to follow-up in this study at the 24 months probably did not differ substantially from those followed. Secondly, there was no statistically significant difference between the two groups in the distribution of children and molars lost to follow-up. The risk of detection bias was not totally avoided in this study. First of all, it is a common problem for studies comparing ART sealant with resin sealant because of the distinctly different appearance of the two types of sealants. Secondly, the two examiners placed a small number of sealants in this study after they had finished all the baseline examinations and when the other two independent dentists were not available. The latter factor is probably not too significant as the first follow-up evaluation took place 6 months after the sealant placement.

Resin sealant placement in this study is regarded as the positive control because its effectiveness in fissure caries prevention has been well established [6]. Over the 24-month study period, incidence of dentine caries in the molars receiving resin sealant was only 3.9%, corresponding to a cumulative molar survival rate of 96% (SE = 0.01). This finding is comparable to that in a similar study conducted in southern China [26] as well as those of other studies [23,27,28]. Noting the background that the prevalence of dental caries in 12-year-old children in the study site was 29.8%, with 66.7% of which nested in the permanent 1st molar [18], such a low incidence of dentine caries may be taken to reflect the effectiveness of the resin sealant in preventing fissure caries in this study.

There was no statistically significant difference regarding the 24-month dentine caries incidence rates as well as the molar survival rates between the two sealant groups in this study. Multivariate two-level logistic regression analysis (GEE modeling) which can account for the effects of confounding factors and data clustering was adopted [29]. Results of the GEE modeling confirmed that the risks to develop dentine caries in the fissures of the molars in the two sealant groups did not significantly differ from each other. Therefore, the null hypothesis of this study cannot be rejected.

There is currently no systematic review specially targeted on the comparison between ART sealant and light-cured resin sealant in fissure caries prevention. A literature search yielded four comparable original studies [16,30-32]. Oba et al. found 3-year caries incidence rates of around 10% in both groups of molars receiving ART sealant and resin sealant respectively [31]. Low 2-year caries incidence rates (<2%) in both the ART sealant and resin sealant groups were found by Chen et al., again, no significant difference in their effectiveness in fissure caries prevention was found [30]. In the third study, no caries was observed in molars receiving ART sealant over 2 years and this was significantly better than that in the resin sealant group which showed a 4% caries incidence rate over the same period [16]. A recent long term study showed 4-year caries incidence rates in both the ART sealant and resin sealant groups of less than 4% with no significant difference in their effectiveness in fissure caries prevention been found between them [32].

The 24-month retention rate of the resin sealant in this study is lower than those commonly reported in other studies which are around 80% [6,33]. The lower retention rate of resin sealants in this study may be related to the less-than-optimal operating conditions for its placement which was the compromised school setting instead of a well-equipped clinical environment. Under field conditions, ample illumination, good moisture control, and thorough cleaning of the pits and fissures cannot be guaranteed. Similar problems were encountered in another study where sealants were also provided in a school setting which found a 93.8% complete loss of resin sealant 3 years after placement [31]. Improvement of the operation conditions would probably lead to a better retention of resin sealants. Despite this, retention of the resin sealants in this study was still significantly higher than that of the ART sealants. This is in line with what has previously been reported [6].

The 24-month retention rate of ART sealant in this study (52% full + partial retention) is generally lower than those reported in previous comparable studies conducted under similar field settings which ranged 50-72% over a longer 3-year period [31,34-36]. It was found in previous studies that retention of ART sealant was influenced by the experience of different operators with experienced ones performed better than the inexperienced ones [15,34,35]. Probably operators in those studies received better training and being more experienced in ART sealant placement.

Although a significantly lower retention rate and shorter retention time than those of the fluoride-releasing resin sealant were found for the ART sealant in this study, the effectiveness of ART sealant in fissure caries prevention did not differ significantly from that of the resin sealant used in this study. This might be explained by the findings of Beiruti et al. that high-viscosity GIC sealants had a four times higher chance of preventing caries development in re-exposed pits and fissures of occlusal surfaces in first molars than resin sealant over a 1- to 3-year period [16]. It is also in agreement with a long-term follow-up study on ART sealant that the drop of the effectiveness of ART sealant in fissure caries prevention lagged the fall of its retention [15]. In that study, it was found that dentine caries in molars with complete loss of ART sealant was relatively infrequent. Probably there were some clinically undetectable glass-ionomer particles retained in the deeper parts of the fissure as observed by Frencken and Wolke [37] and these offered continuing protection against caries. It is reiterated that due to the difference in the materials used, the success of a fissure sealing method should finally be assessed by the outcome of dentine caries prevention rather than material retention [9].

In the final GEE modeling in this study, it was also found that presence of early caries in the occlusal surfaces of the molars before sealant placement and shorter retention of sealant on the molars significantly increased the risk of developing dentine caries in the pits and fissures. These findings are consistent with those of other studies [1,6,26,38].

Comparing the placement of the two types of sealants, it is noted that the number and training of dental personal required as well as the time used are similar. However, the set-up and running of an ART sealant program for children in schools will be easier than those of a resin sealant program. It is because the equipment required for ART sealant placement is rather simple (only a few hand instruments) whereas that for resin sealant placement requires an electrically powered dental unit. Given that the two methods yield similar effectiveness in fissure caries prevention, their relative affordability, availability, and simplicity should be considered when making a choice between the two. ART sealant might be more appropriate than resin sealant for use in less developed areas or in outreach dental service programs. In addition, the use of ART sealant instead of resin sealant will prevent the harmful exposure of children to additional Bisphenol A (BPA) releasing materials since systematic review has shown that BPA can be released from resin-based pit and fissure sealant [39]. BPA exhibits a variety of toxicity effects on human bodies and evidences on the relationship between BPA and some adverse human health conditions (e.g. obesity, diabetes, coronary heart disease, enamel defects) have been shown by various studies [40,41]. The raised safety concern has forced FDI to release a policy statement which discouraged the use of BPA in dental materials [42].

## Conclusions

Within the limitation of this study, it is concluded that though the retention of fluoride-releasing resin sealant was better than that of the ART sealant, their effectiveness in preventing fissure caries in permanent molars did not differ significantly over 24 months. ART sealants could be a good alternative when and where resources for resin sealant placement are not readily available.

## Abbreviations

ART: Atraumatic restorative treatment; GIC: Glass ionomer cement; SDF: Silver diammine fluoride solution; NaF: Sodium fluoride varnish.

## Competing interests

The authors declare that they have no competing interests.

## Authors’ contributions

BL helped the carrying out of the study and prepared the manuscript. YX participated in the study’s design, performed the study as well as assisted the preparation of the manuscript. EL and CC participated in the study’s design, supervised the carrying out of this study, and read and approved the final manuscript.

## Pre-publication history

The pre-publication history for this paper can be accessed here:

http://www.biomedcentral.com/1472-6831/14/54/prepub
